# Efficacy and safety of different antithrombotic treatment regimens in patients undergoing transcatheter mitral valve repair and atrial fibrillation; A meta-analysis and systematic review

**DOI:** 10.3389/fcvm.2026.1743097

**Published:** 2026-07-21

**Authors:** Fuli Zhu, Zihan Yu, Zhe He

**Affiliations:** 1 Department of Cardiology, Beijing Luhe Hospital, Capital Medical University, Beijing, China; 2 Department of Clinical Medicine, Chengde Medical University, Chengde City, Hebei, China

**Keywords:** antithrombotic therapy, atrial fibrillation, efficacy and safety, meta-analysis, transcatheter mitral valve repair

## Abstract

**Background:**

Optimal antithrombotic therapy following transcatheter mitral valve repair (TMVR/TEER) in patients with atrial fibrillation remains uncertain, with substantial practice variation and reliance on nonrandomized data.

**Methods:**

We performed a systematic review and network meta-analysis of observational studies evaluating antithrombotic strategies after TMVR/TEER with atrial fibrillation. Antithrombotic regimens were harmonized into two main categories: oral anticoagulation [OAC; including vitamin K antagonists [VKAs] and direct oral anticoagulants [DOACs]] and antiplatelet therapy (APT; including aspirin or clopidogrel, alone or in combination). Outcomes of interest were ischemic stroke, major bleeding, and all-cause mortality. Pairwise and network random-effects models were applied to derive odds ratios (ORs) with 95% confidence intervals (CIs).

**Results:**

Four multicenter observational cohorts (*n* = 2,098) were included. Across comparisons, OAC strategies demonstrated a favorable profile compared with APT for bleeding risk reduction, while DOACs showed heterogeneous results depending on comparator drug and study region (phenprocoumon in Europe vs. warfarin in the U.S.). Mortality findings consistently favored OAC (particularly DOACs) potential benefits over APT. For stroke, OAC (phenprocoumon) showed better ischemic prevention, indicating more effectiveness in minimizing stroke risk compared with APT. Network geometry was limited, with most contrasts informed by only one or two studies.

**Conclusions:**

In patients undergoing TMVR/TEER with atrial fibrillation, OAC strategies appeared to be associated with lower bleeding risk and potentially favorable mortality outcomes compared with antiplatelet therapy. However, conclusions regarding comparative efficacy, particularly for stroke prevention, should be interpreted cautiously due to the limited number of studies and the observational nature of the available evidence. Thus, the evidence base remains limited by observational design, heterogeneous comparators, and inconsistent outcome definitions. Randomized trials are urgently needed to guide antithrombotic management in this high-risk population.

## 1 Introduction

Transcatheter mitral valve repair (TMVR), particularly via edge-to-edge techniques such as Mitra-Clip (TEER), has emerged as a vital therapeutic option for patients with severe mitral regurgitation who are considered high risk for conventional surgery. In the United States alone, over 5,600 TMVR procedures were performed between 2013 and 2016, highlighting its growing adoption in clinical practice (1). In patients undergoing TMVR, approximately 56%–73% had preexisting atrial fibrillation [73.3% in a multi-center cohort (2); pooled prevalence ∼56%–63% in a systematic review of Mitra-Clip studies 3].

Patients with atrial fibrillation (AF) undergoing transcatheter mitral valve repair (TMVR), often via edge-to-edge techniques, face complex therapeutic dilemmas. AF is common in this population, with registries reporting prevalence rates exceeding 60%–75% among TMVR recipients (4). These individuals are exposed to elevated risk for thromboembolic events, including stroke and device-related thrombosis, due to factors such as atrial stasis, endothelial injury from device placement, and a hypercoagulable state from comorbid conditions. At the same time, many are elderly with multiple comorbidities, which heightens bleeding risk post-procedure. This delicate balance between thrombosis prevention and bleeding avoidance underscores the critical need for evidence-based antithrombotic strategies in this setting.

Despite the prevalence of AF and clear thromboembolic risk, no standardized antithrombotic protocol exists post-TMVR. Current practice tends to follow empirical models, such as early post-TMVR dual antiplatelet therapy (DAPT) for 1–6 months or longer in those without anticoagulation indications, and oral anticoagulation (OAC), often with vitamin K antagonists (VKA) or select novel oral anticoagulants (DOACs) considered when AF is present (5). These approaches are based on limited observational findings and guidelines extrapolated from surgical bioprosthetic mitral valve data. Early meta-analysis suggests that OAC, especially when used alone, may reduce stroke rates without significantly increasing bleeding risk, compared to no anticoagulation or combined therapy, though evidence quality remains limited (6).

Accordingly, this meta-analysis aims to synthesize the available observational cohorts to quantify the efficacy and safety of antithrombotic regimens post-TMVR in patients with AF. By comparing outcomes such as stroke, major bleeding, and all-cause mortality across different antithrombotic strategies, we hope to identify regimens that offer optimal net clinical benefit. The findings will provide much-needed clarity to clinical decision-making, support guideline development, and highlight knowledge gaps warranting future investigation.

## 2 Materials and methods

### 2.1 Data sources and strategy

A systematic search of PubMed, Web of Science was performed up to August 25, 2025 by 2 of the authors Fuli Zhu & Zihan Yu to ensure screening, utilizing a combination of free-text and subject terms. The search strategy was tailored to each database; each string was applied using terms like “antithrombotics”, “mitral valve repair”, “atrial fibrillation”, to ensure a wide retrieval of papers.

**Figure 1:**
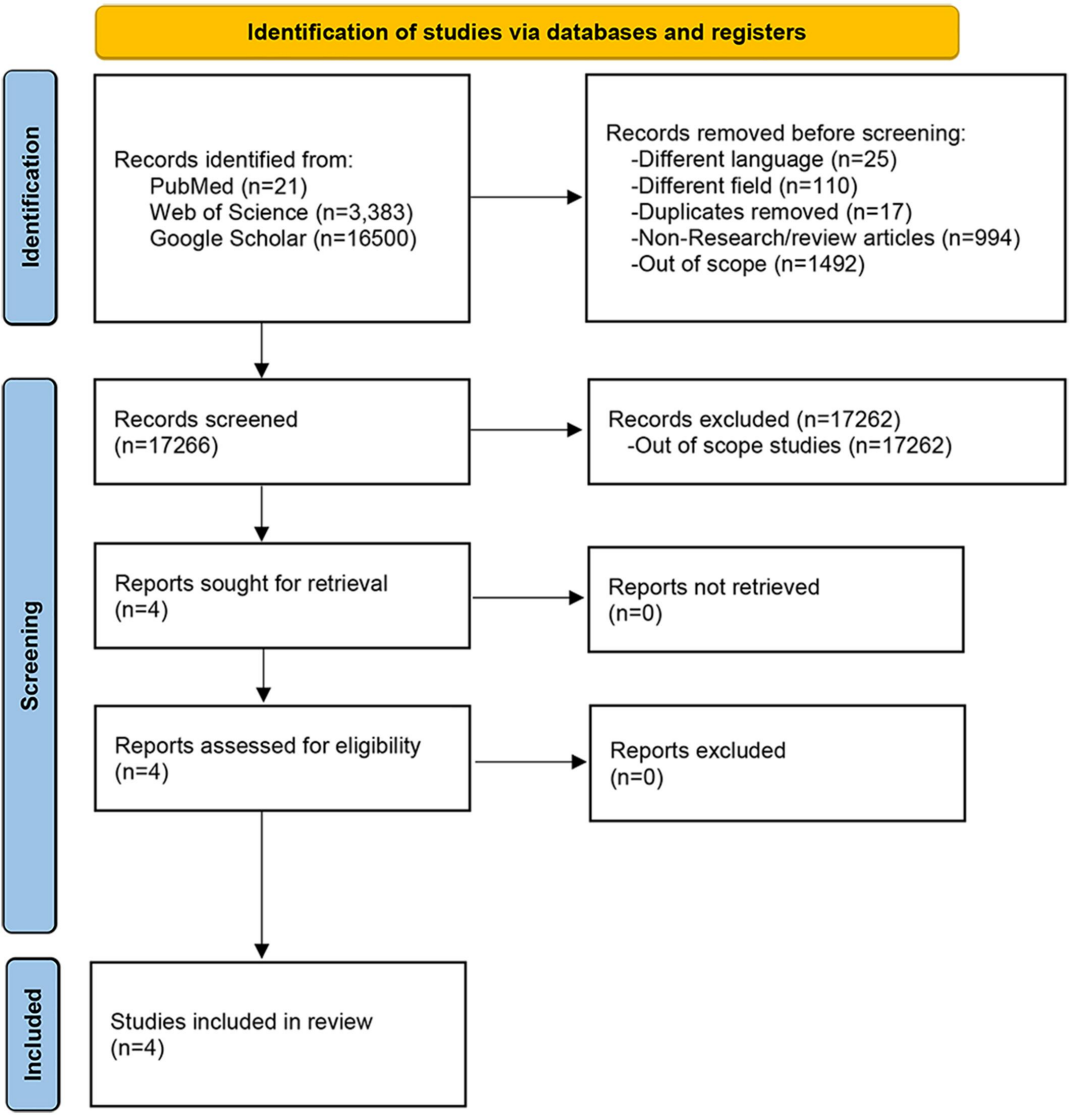
Prisma flow diagram.

The final screening was done via a complementary search in Google Scholar. The studies retrieved and screened are shown in Table 1 and Figure 1. Any issues or conflicts found in screening were resolved by a third author Zhe He.

**Table 1:** Search strategy.

Database	Search Strategy	Results
PubMed	(antithrombotic OR Warfarin OR rivaroxaban OR edoxaban OR apixaban OR dabigatran OR clopidogrel OR Aspirin) AND (transcatheter mitral valve repair OR transcatheter mitral valve edge to edge repair OR TEER OR TMVR OR Mitraclip) AND atrial fibrillation	21
Web of Science	(antithrombotic OR anticoagulant OR antiplatelet OR warfarin OR aspirin) AND (“transcatheter mitral valve repair” OR TMVR OR TEER OR MitraClip)	3,383
Google scholar	(antithrombotic OR Warfarin OR rivaroxaban OR edoxaban OR apixaban OR dabigatran OR clopidogrel OR Aspirin) AND (transcatheter mitral valve repair OR transcatheter mitral valve edge to edge repair OR TEER OR TMVR OR Mitraclip) AND atrial fibrillation	16,500

### 2.2 Data extraction and quality assessment

**Figure 2:**
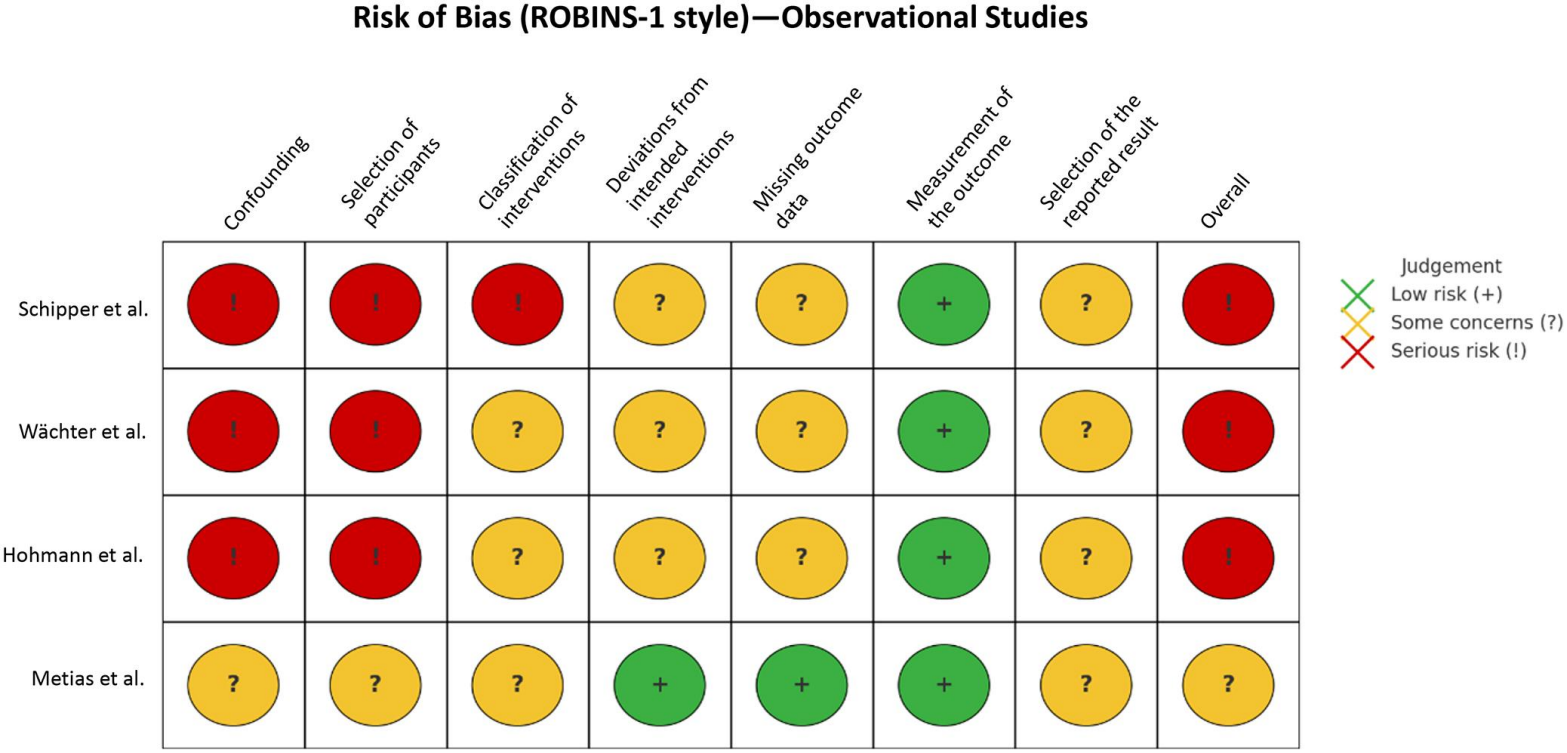
Risk of bias summary.

The quality of the included studies was assessed using the appropriate Cochrane Risk of Bias tools (RoB2.0 for randomized trials and ROBINS-I for observational studies) (Figure 2) as shown across the observational studies included, most were judged at serious risk of bias, mainly due to residual confounding, potential selection bias, and incomplete follow-up. Schipper et al., Waechter et al., and Hohmann et al. were each rated at serious risk, with concerns relating to nonrandomized treatment allocation, substantial exclusions, and possible misclassification of treatment exposure. Mentias et al. applied advanced statistical adjustments, including inverse probability weighting and propensity score matching, resulting in an overall judgment of moderate risk of bias. In all studies, the risks related to outcome measurement were considered low, as the endpoints were hard clinical events such as mortality, stroke, or major bleeding. However, the lack of trial registration or predefined analysis plans raised moderate concerns about selective reporting. Overall, the evidence base was characterized by varying levels of risk, but the predominance of observational designs limited the certainty of causal inference. For additional measure, the quality of the included observational studies was assessed based on the STROBE (Strengthening the Reporting of Observational Studies in Epidemiology) checklist to ensure transparent and comprehensive reporting (Supplementary Table S1).

Regimens were classified into broad categories rather than maintaining the heterogeneous subclassifications reported in individual cohorts. Therapies were grouped as oral anticoagulation (OAC) or antiplatelet therapy (APT), given that the use of direct oral anticoagulants (DOACs), vitamin K antagonists (VKAs), single antiplatelet therapy (SAPT), and dual antiplatelet therapy (DAPT) varied between studies and patient sub-groups. Under OAC, the included agents were DOACs such as rivaroxaban, apixaban, edoxaban, and dabigatran (Schipper et al., Mentias et al.), as well as phenprocoumon, the predominant VKA used in Germany and the Netherlands (Schipper et al., Hohmann et al.) as well as warfarin. APT comprised aspirin and/or clopidogrel, administered either as single antiplatelet therapy (SAPT) or dual antiplatelet therapy (DAPT). The specific regimen, intensity, and duration of antiplatelet treatment varied substantially across studies, introducing heterogeneity within the pooled APT category (Hohmann et al., Wächter et al.). By harmonizing these regimens into OAC and APT categories, we minimized mis-classification bias and facilitated meaningful comparisons across the available evidence base. Major bleeding events were defined according to the criteria used in the original studies, including ISTH, BARC, or TIMI definitions. Because outcome reporting was limited and heterogeneous across studies, we accepted these definitions to maximize data inclusion while acknowledging the potential for variability in bleeding classification.

### 2.3 Selection criteria

#### 2.3.1 Inclusion criteria

- Adult patients (≥18 years) undergoing transcatheter mitral valve edge-to-edge repair (TEER).
- Documented history of atrial fibrillation requiring anticoagulation.
- Treatment with one of the following regimens post-procedure:
  - Oral anticoagulants (OAC, including DOACs)
  - Vitamin K antagonists (VKA phenprocoumon)/Warfarin
  - Antiplatelet therapy (APT), alone or in combination.
  - major bleeding was defined per study
- Studies reporting at least one clinical outcome of interest: ischemic stroke/systemic embolism, all-cause mortality, or major bleeding (intracranial or gastrointestinal)

#### 2.3.2 Exclusion criteria

- Patients without atrial fibrillation or not requiring anticoagulation.
- Surgical mitral valve replacement (non-transcatheter)
- Case reports, reviews, editorials, or studies lacking extractable arm-level data.
- Studies without outcome reporting are relevant to ischemic, bleeding, or survival endpoint.

Selected Studies and extracted outcomes are showed in Table 2.

**Table 2:** Study outcomes.

Study		STROKE				DEATH				BLEEDING EVENT			
	*N*=	APT	OAC	VKAp	WAR	APT	OAC	VKAp	WAR	APT	OAC	VKAp	WAR
Mentias 2022	DOAC = 491		20/491		34/687		69/491		131/687		54/491		103/687
	WAR = 687												
Hohmann 2022	APT = 458					31/458	37/540			16/458	8/540		
	OAC = 540												
Waechter 2022	OAC = 146	0/149	1/146			6/149	4/146			5/149	3/146		
	APT = 149												
Schipper 2025	DOAC = 61		7/61	5/145							9/61	9/145	
	VKA = 145												

### 2.4 Statistical analysis

The primary outcomes were stroke, all-cause mortality, and bleeding events, each analyzed separately. For every direct comparison, odds ratios (ORs) with 95% confidence intervals (CIs) were calculated as the effect size using the observed events and denominators from each study. Continuity corrections were applied for zero-event cells.

A pairwise meta-analysis was first performed for each available head-to-head comparison using the random-effects model (DerSimonian-Laird estimator) to provide conventional pooled estimates. Between-study heterogeneity was assessed using the Q statistic and *I*^2^ statistic, with *I*^2^ ≤ 50% or *P* ≥ 0.1 considered low heterogeneity, in which case a fixed-effects model was reported; otherwise, a random-effects model was retained. Sensitivity analyses were planned to sequentially exclude studies with high impact on heterogeneity.

A network meta-analysis (NMA) was subsequently performed within a frequentist framework using R software. The analysis used the Paule–Mandel estimator for the between-study variance (*τ*^2^) and inverse-variance weighting to combine treatment contrasts. OAC was defined as the reference node. Treatments included antiplatelet therapy (APT), oral anticoagulation (OAC), and vitamin K antagonists. Multi-arm trials were not present; all the studies included were two-arm designs.

Relative treatment effects were estimated for each pairwise contrast across the network, expressed as ORs with 95% CIs. Results were summarized in league tables and visually represented in pictorial heatmaps, where values <1 favored the row treatment. P-scores (analogous to SUCRA in Bayesian NMA) were calculated to rank treatments from most to least effective/safe for each outcome. Higher P-scores indicated greater probability of being the best regimen for reducing adverse events.

Network geometry was depicted with nodes sized by patient sample size and edges weighted by number of studies per comparison. Evidence connectivity revealed OAC as the central comparator, directly linked to both APT and VKA, while APT–VKA comparisons were exclusively indirect.

All analyses were conducted in Python for data structuring, geometry visualization, and pairwise pooling, and in Rstudio 4.5.1 (net meta) for the NMA implementation and heatmap visualization.

## 3 Results

### 3.1 Study characteristics

Across the four included observational studies [Mentias et al. (11), Hohmann et al. (7), Wächter et al. (2), and Schipper et al. (9)], baseline patient characteristics were generally comparable, although some variability was observed depending on study design and treatment allocation. The study populations were uniformly elderly, with mean ages ranging from approximately 74–79 years. Women represented an important proportion of participants, with female inclusion varying between 34% and 49% across treatment groups, the highest proportion being reported in Mentias et al. See Supplementary Table S2.

Comorbidities were highly prevalent, particularly hypertension, which was reported in more than 80% of patients across most series. Baseline stroke risk was consistently high, as reflected by CHA₂DS₂-VASc scores that typically ranged from 4 to 6. This underscores the elevated thromboembolic burden in these cohorts. Collectively, the included populations were elderly, predominantly hypertensive, and at substantial risk of ischemic and bleeding events. These common features established a clinically relevant foundation for comparing the safety and effectiveness of oral anticoagulation, vitamin K antagonists, direct oral anticoagulants, and antiplatelet therapies in the post-TMVR setting.

### 3.2 Quality of included studies

Risk of bias assessments revealed 3 cohorts showing serious limitations related to confounding, selection bias, and treatment misclassification, whereas the fourth study, which used propensity score matching and inverse probability weighting, was adjudi-cated at moderate risk (Figure 2). In all studies, outcome ascertainment (ischemic stroke, bleeding, mortality) was considered reliable due to hard clinical endpoints, though moderate concerns remained regarding selective reporting, as few studies were preregistered.

### 3.3 Sensitivity analysis

We performed multiple sensitivity analyses using Python V3.11. First, fixed- and random-effects models produced identical pooled estimates (*τ*^2^ = 0 in all meta-analyses with ≥2 studies). For APT vs. OAC, pooled odds ratio (OR) for bleeding favoured OAC (OR = 2.18 for APT vs. OAC; 95% CI 1.04–4.57; *k* = 2). Leave-one-out showed this result was driven largely by Wächter 2022: removing Wächter yielded OR = 1.66 (0.39–7.06), while removing Hohmann 2022 retained significance [OR = 2.41 (1.02–5.68)]. For mortality (APT vs. OAC), results were neutral overall (OR = 1.04; 0.66–1.65; k = 2) and remained non-significant in leave-one-out analyses (0.99–1.49). TEER stroke data com-paring APT vs. OAC were available from one study only (Wächter 2022) and were imprecise (OR = 0.32; 0.01–8.03). Among OAC class comparisons, single-study estimates showed lower bleeding (OR = 0.70; 0.49–0.996) and lower mortality (OR = 0.69; 0.51–0.95) with DOACs vs. warfarin (Mentias 2022), with no significant difference in stroke (OR = 0.82; 0.46–1.43). In Schipper 2025, DOAC vs. VKA (phenprocoumon) yielded imprecise single-study estimates (bleeding OR = 2.62; 0.98–6.95; stroke OR = 3.63; 1.10–11.93) based on small numbers. Overall, the direction of effect was robust for the APT vs. OAC bleeding outcome but other contrasts were limited by single-study evidence and wide confidence intervals.

### 3.4 Pairwise meta-analysis

See Table 3.

**Table 3:** Pairwise meta-analysis of individual outcomes and the anticoagulants.

Outcome	Comparison	OR	95% CI	k (studies)
Bleeding	APT vs OAC	2.18	1.044–4.571	2 (Hohmann et al, Waechter Et al)
Bleeding	DOAC vs VKAp	2.62	0.984–6.952	1 (Schipper et al)
Bleeding	DOAC vs Warfarin	0.70	0.493–0.996	1 [Mentias et al. (11)]
Death	APT vs OAC	1.04	0.656–1.651	2 (Hohmann et al, Waechter Et al)
Death	DOAC vs Warfarin	0.69	0.505–0.954	1 [Mentias et al. (11)]
Stroke	APT vs OAC	0.32	0.013–8.029	1 (Waechter Et al)
Stroke	DOAC vs VKAp	3.63	1.104–11.929	1 (Schipper et al)
Stroke	DOAC vs Warfarin	0.82	0.464–1.435	1 [Mentias et al. (11)]

DOAC, Direct oral anticoagulants; OAC, Oral Anticoagulant; VKAp, Vitamin k antagonist phenprocounom; WAR, Warfarin; APT, Antiplatelet therapy.

#### 3.4.1 Stroke

**Figure 3:**
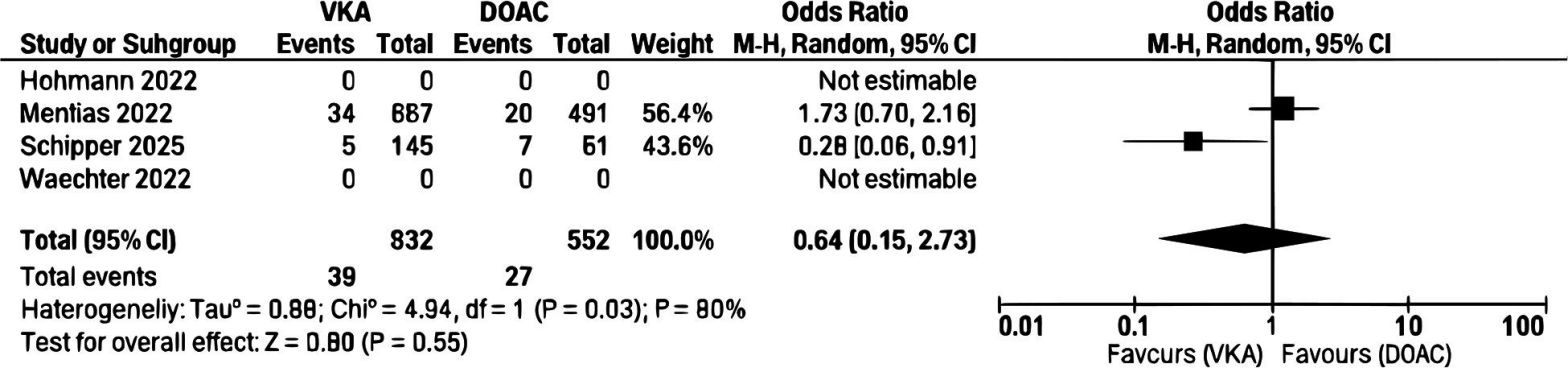
Forest plot for pairwise meta-analysis for stroke.

The stroke analyses were based on very limited evidence, with each comparison informed by a single observational study and relatively few events, resulting in wide confidence intervals and substantial statistical uncertainty. APT showed a nonsignificant trend toward lower stroke risk compared with OAC (OR 0.32, 95% CI 0.01–8.03; 1 study), though this result was based on a single, underpowered study and inconsistent with broader evidence favoring OAC. The comparison of DOAC vs. VKA phenprocoumon demonstrated a higher risk of stroke with DOAC (OR 3.63, 95% CI 1.10–11.93; 1 study). In contrast, DOAC vs. warfarin (US) indicated no significant difference between groups (OR 0.82, 95% CI 0.46–1.44; 1 study). Overall, the stroke analyses were underpowered and showed inconsistent directions of effect across comparisons. When pooling all VKA comparators (including phenprocoumon and warfarin), DOAC was not significantly different from VKA for ischemic stroke (pooled OR 0.64, 95% CI 0.15–2.73; 2 studies, random-effects), though heterogeneity was substantial (*I*^2^ = 80%). (Figure 3).

#### 3.4.2 Death

**Figure 4:**
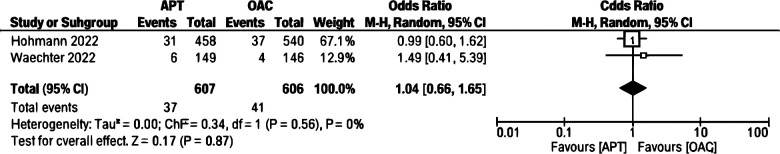
Forest plot for pairwise meta-analysis for all-cause mortality (death).

For all-cause mortality, results were more consistent. APT yielded a nonsignificant trend toward higher mortality risk compared with OAC (OR 1.04, 95% CI 0.66–1.65; 2 studies) (Figure 4). However, DOAC vs. warfarin (US) demonstrated a significant reduction in mortality favoring DOAC (OR 0.69, 95% CI 0.51–0.95; 1 study). Taken together, these findings indicate potential mortality benefits of DOAC compared with warfarin, while APT offers no clear advantage over OAC.

#### 3.4.3 Bleeding

**Figure 5:**
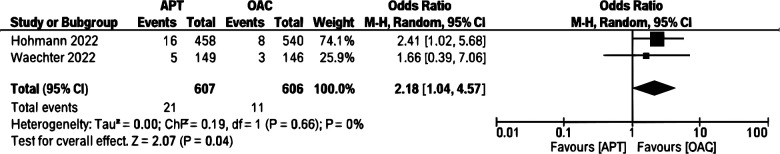
Forest plot for pairwise meta-analysis for bleeding.

In terms of bleeding complications, APT was associated with a significantly higher risk compared with OAC (OR 2.18, 95% CI 1.04–4.57; 2 studies) (Figure 5). DOAC vs. VKA phenprocoumon showed a nonsignificant trend toward increased bleeding with DOAC (OR 2.62, 95% CI 0.98–6.95; 1 study). Conversely, DOAC vs. warfarin (US) suggested a protective effect of DOAC with borderline significance (OR 0.70, 95% CI 0.49–1.00; 1 study). These results collectively indicate heterogeneity in bleeding outcomes depending on the comparator, but a consistent signal of higher bleeding risk with APT. The divergent findings may reflect differences in patient populations and VKA agents across regions (phenprocoumon in Europe vs. warfarin in the US).

### 3.5 Network meta-analysis

**Figure 6:**
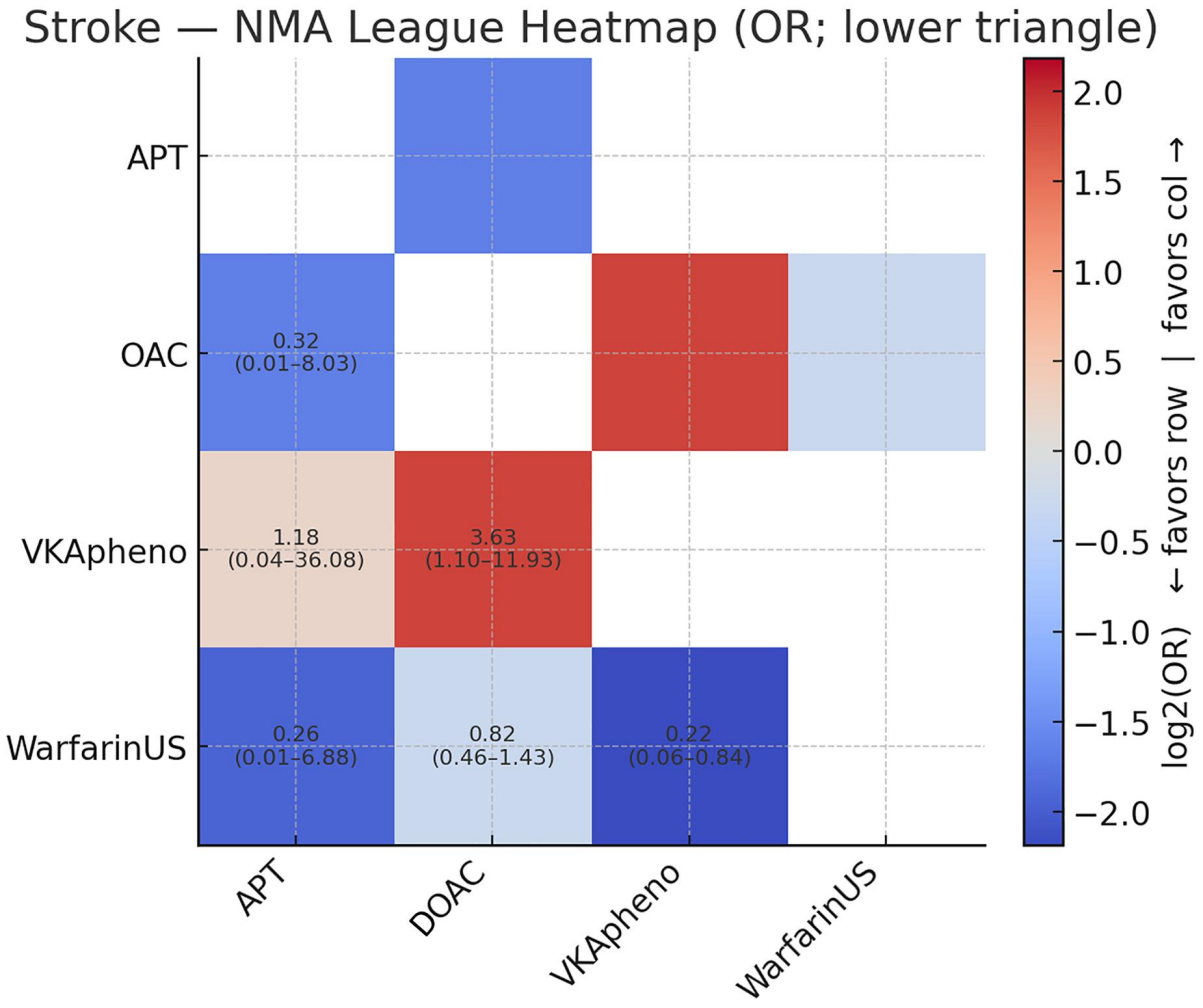
Heatmap rankings for stroke.

**Figure 7:**
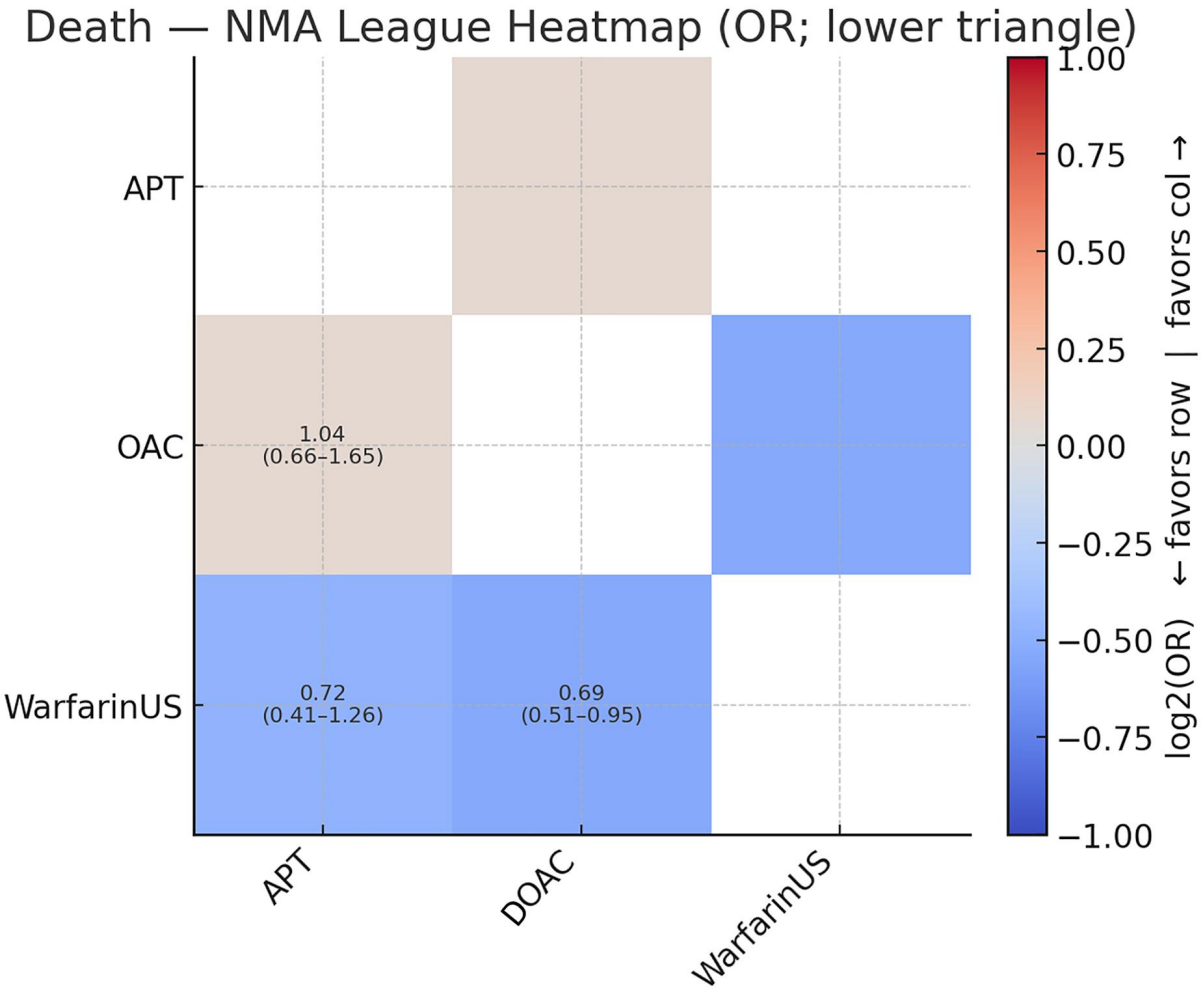
Heatmap rankings for all-cause mortality.

**Figure 8:**
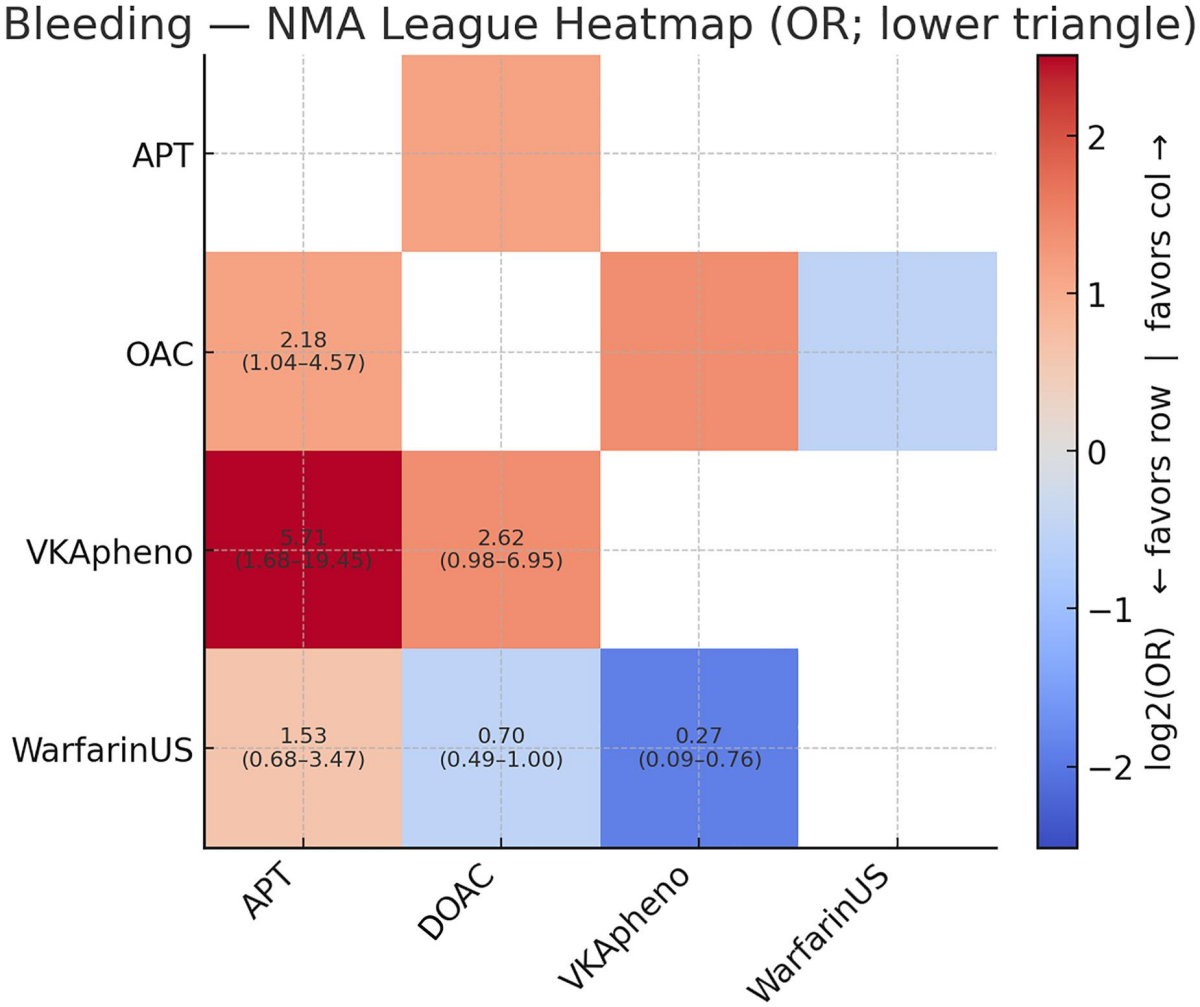
Heatmap rankings for bleeding outcomes.

The network meta-analysis (NMA) evaluated the comparative effectiveness and safety of antithrombotic strategies, including APT, DOAC, VKA phenprocoumon, and standard warfarin (WarfarinUS), across three key outcomes: stroke, death, and bleeding. League heatmaps (Figures 6–8) summarize pairwise comparisons using ORs on a log₂ scale, where blue shades indicate reduced odds (favoring row treatment) and red shades indicate increased odds (favoring column treatment).

#### 3.5.1 Stroke

In the NMA for stroke (Figure 6), significant heterogeneity was observed among treatment regimens. In the network analysis, DOAC appeared to have a higher estimated stroke risk compared with phenprocoumon; however, this estimate was derived from a single observational study with limited events and wide confidence intervals and therefore should not be interpreted as definitive evidence of a true treatment difference. The values (OR = 3.63, 95% CI 1.10–11.93) indicate a relative disadvantage of DOAC in this comparison. Compared to WarfarinUS, VKA phenprocoumon demonstrated a lower stroke risk (OR = 0.22, 95% CI 0.06–0.84), supporting its higher efficacy. Comparisons between OAC and APT (OR = 0.32, 95% CI 0.01–8.03) and between WarfarinUS and DOAC (OR = 0.82, 95% CI 0.46–1.43) were not statistically significant, suggesting comparable performance between these regimens.

#### 3.5.2 Death

DOAC demonstrated a significant survival advantage over WarfarinUS (OR = 0.69, 95% CI 0.51–0.95) and OAC showed a nonsignificant similar trend toward lower mortality risk compared with APT (OR = 1.04, 95% CI 0.66–1.65). Comparison between APT and WarfarinUS (OR = 0.72, 95% CI 0.41–1.26) did not reach statistical significance, suggesting similar survival between these regimens. Overall, DOAC was associated with the most favorable survival profile (Figure 7).

#### 3.5.3 Bleeding

In the NMA assessing bleeding risk (Figure 8), APT was associated with a significantly higher bleeding risk compared with OAC (OR = 2.18, 95% CI 1.04–4.57) and a substantially higher risk compared with VKA phenprocoumon (OR = 5.21, 95% CI 1.08–17.35). DOAC exhibited higher bleeding odds than VKA phenprocoumon (OR = 2.62, 95% CI 0.98–6.95). consistently, VKA phenprocoumon showed the lower bleeding risk over WarfarinUS (OR = 0.27, 95% CI 0.09–0.76). DOAC showed a marginally lower risk compared with WarfarinUS (OR = 0.70, 95% CI 0.49–1.00). These findings suggest a higher bleeding risk associated with the aggregated APT group in the included studies; however, this observation should be interpreted cautiously because the APT category included heterogeneous regimens such as SAPT and DAPT with varying treatment durations.

Overall, the NMA consistently showed OAC was a favorable efficacy and safety profiles, being associated with reduced risks of bleeding and mortality compared with APT. Further, DOAC demonstrated more potential benefits for mortality compared to Warfarin. It also highlights possible differences between European and U.S. VKA regimens, with phenprocoumon more favorable for bleeding outcomes, indicating limited clinical advantage of Warfarin in this setting, though uncertainty remained wide. The confidence intervals across most contrasts underscore the limited power of the available evidence, requiring cautious interpretation.

### 3.6 Treatment rankings

To complement the pairwise and network meta-analysis findings, we conducted ranking analyses to provide a probabilistic interpretation of the relative efficacy and safety of the different anticoagulant strategies. The league tables and corresponding heatmaps (Figures 6–8) were first generated from the network meta-analysis, displaying odds ratios and confidence intervals for each treatment comparison across outcomes. These visualizations allowed us to identify the relative direction and magnitude of effects, but they did not directly convey the likelihood of each treatment being the best, second-best, or worst option.

To address this, we constructed Bayesian-inspired ranking bar plots using Monte Carlo sampling from the point estimates and uncertainty bounds provided in the league tables. This method approximates the rank probabilities by repeatedly simulating treatment contrasts from the observed odds ratios and their 95% confidence intervals, thereby estimating how frequently each treatment would occupy a given rank across iterations. While this approach does not replace full Bayesian posterior sampling, it mirrors the principles of Markov Chain Monte Carlo (MCMC) that underpin standard ranking analyses in network meta-analysis and provides a transparent visualization of ranking uncertainty.

**Figure 9:**
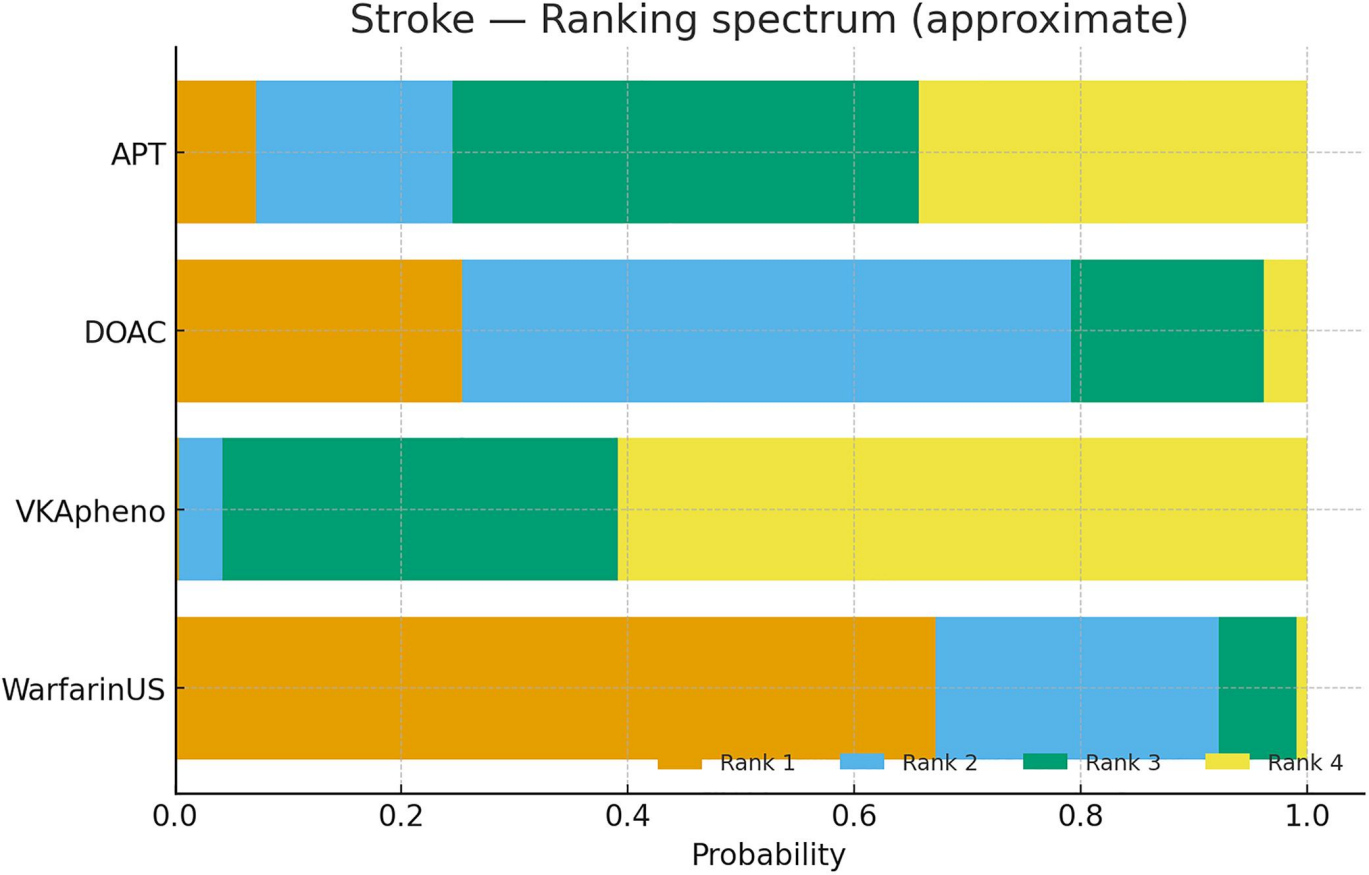
Ranking plot of stroke in first line treatment effects.

**Figure 10:**
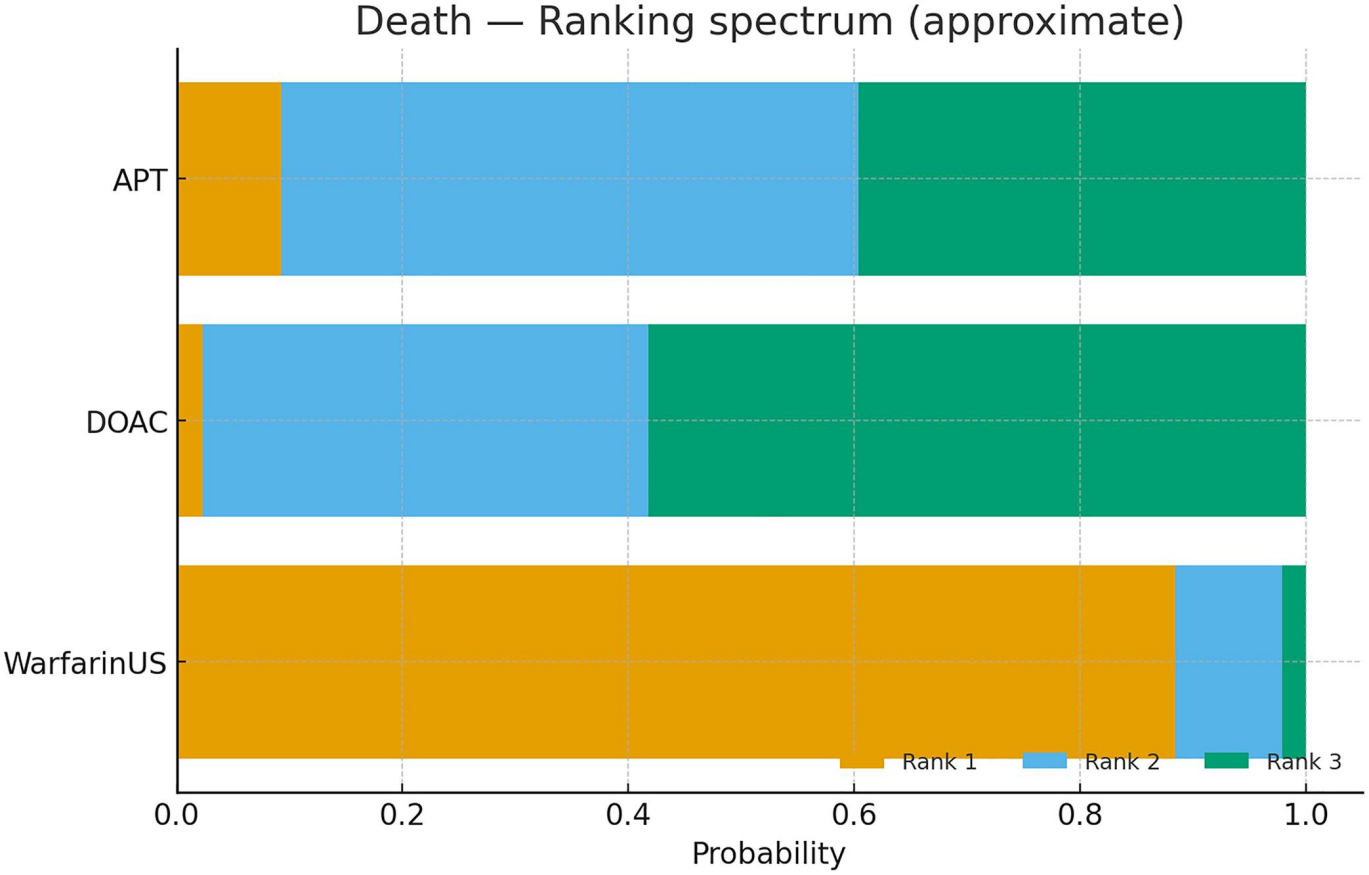
Ranking plot of all-cause mortality (death) in first line treatment effects.

**Figure 11:**
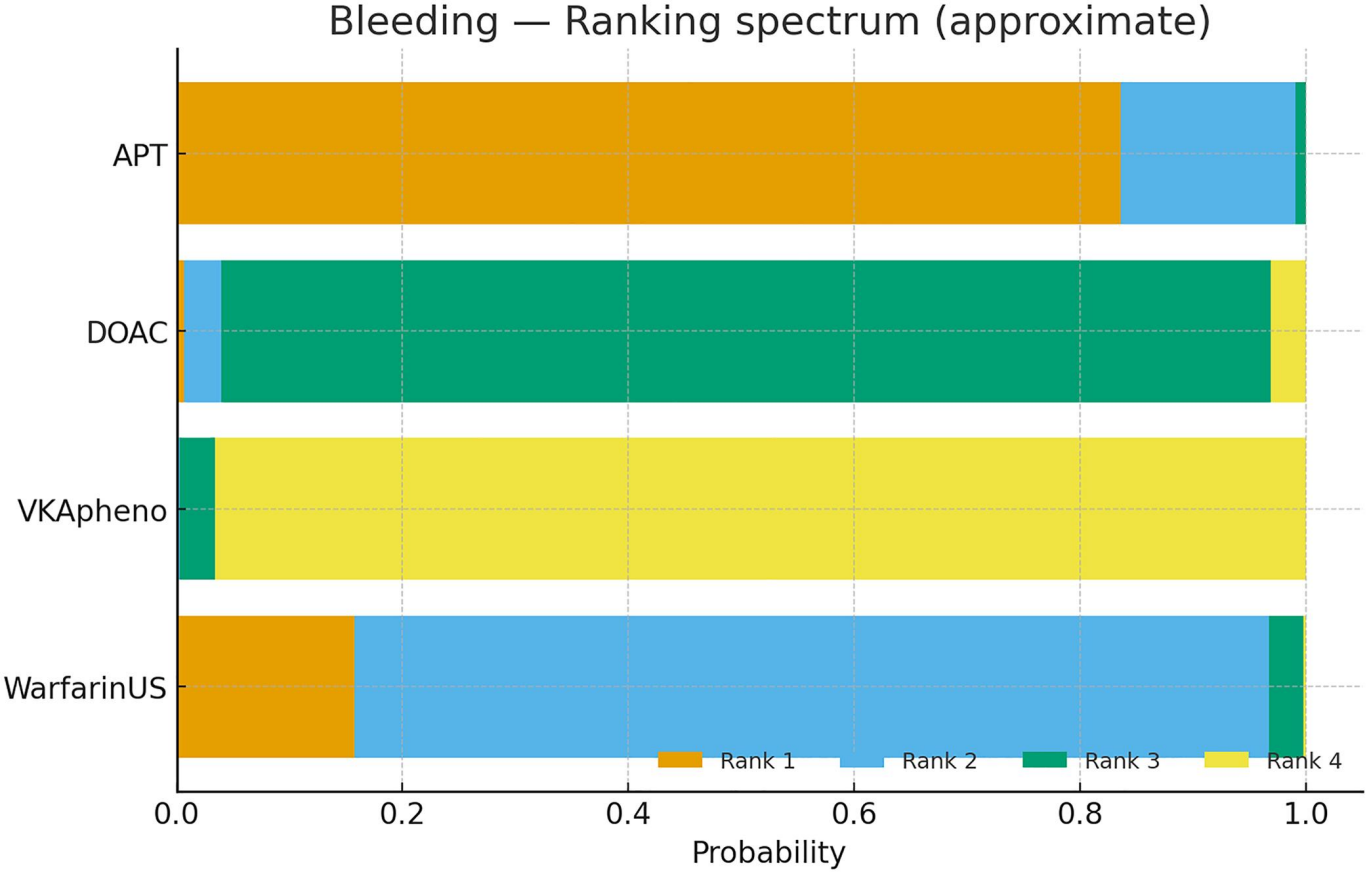
Ranking plot of bleeding events (intracranial + gastroIntestinal bleeds) in first line treatment effects.

Figures 9–11 present the ranking spectra for stroke, death, and bleeding outcomes, illustrating the probability of each treatment achieving a specific rank based on net-work meta-analysis estimates. Higher rank probabilities (Rank 4) correspond to the most favorable efficacy or safety profile, while lower ranks (Rank 1) indicate the least favorable outcomes.

#### 3.6.1 Stroke

As shown in Figure 9, VKA phenprocoumon demonstrated the highest probability of being ranked as the most protective, followed by antiplatelet therapy, with DOAC and Warfarin (U.S. cohorts) more often occupying lower ranks.

#### 3.6.2 Death

In the ranking for all-cause death (Figure 10), DOAC exhibited the stronger likelihood of occupying Rank 3, confirming its favorable survival profile. APT and WarfarinUS showed intermediate to lower ranking probabilities, with WarfarinUS most often appearing in Rank 1, suggesting a comparatively lesser benefit in mortality reduction.

#### 3.6.3 Bleeding

For bleeding risk (Figure 11), antiplatelet therapy ranked most unfavorably, with being Rank 1, denoting the highest bleeding risk among all regimens. In contrast, VKA phenprocoumon exhibited the lowest bleeding risk of occupying Rank 4, consistent with its reduced bleeding odds in the NMA. DOAC followed of most being occupied Rank 4 and 3, suggesting a relative safety advantage in this domain.

Across all ranking spectra, OAC, particularly DOAC, consistently achieved the most favorable probability distributions, occupying Rank 4 or 3 across stroke, bleeding and death outcomes. Conversely, APT demonstrated the poorest overall rankings, highlighting its relative inferiority in both efficacy and safety domains. These ranking results should be interpreted as exploratory and hypothesis-generating rather than definitive evidence of treatment superiority, given the sparse and irregular evidence network underlying the analysis.

**Figure 12:**
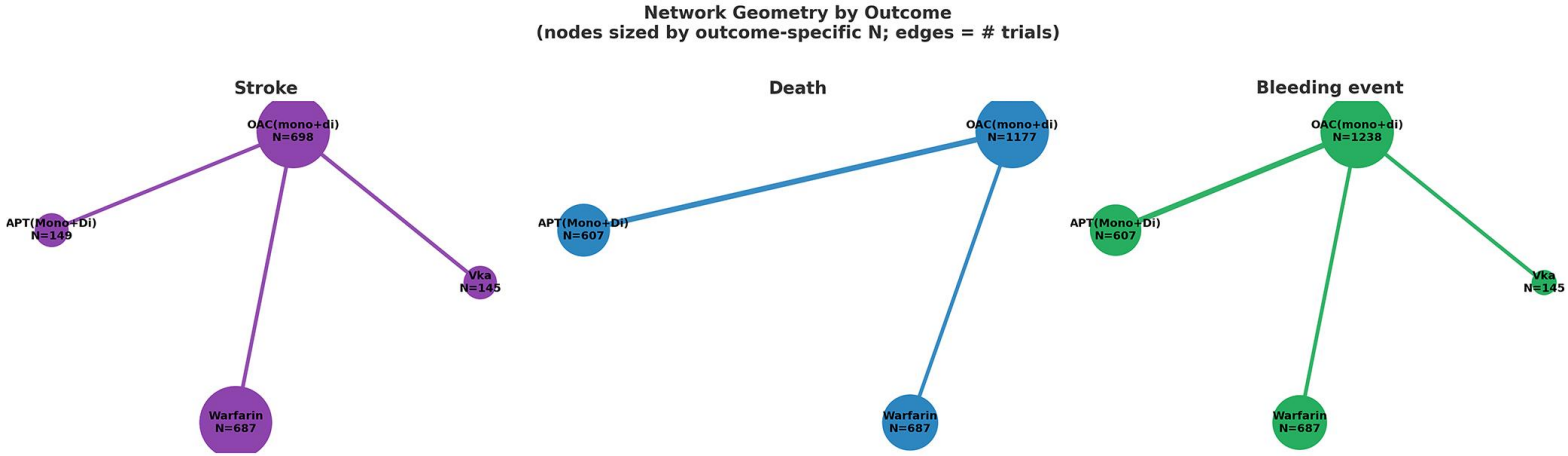
Network plots for endpoints of multiple regimens for post mitral valve repair anticoagulation in patients with atrial fibrillation.

Network geometry plots for stroke, death, and bleeding outcomes illustrate the direct pairwise comparisons among treatments, with node size reflecting sample size and edge thickness proportional to the number of trials. (Figure 12).

## 4 Discussion

This is the first network meta-analysis to synthesize comparative antithrombotic strategies specifically after TMVR/TEER in patients with atrial fibrillation, bringing together heterogeneous real-world cohorts and administrative data into a single evidence framework. Prior reports describe wide practice variation (7) and largely nonrandomized evidence (8), but none has compared all relevant strategies head-to-head within a unified network for this population (9), underscoring the value, and the interpretive care, of our approach.

In the ranking analysis, oral anticoagulation strategies generally appeared to perform more favorably across several outcomes, while antiplatelet therapy tended to rank lower. However, because the network structure was sparse and most comparisons were informed by single observational studies, these ranking results should be interpreted cautiously and considered hypothesis-generating rather than definitive. The findings suggest greater bleeding and death risk with APT compared to OAC. For example, earlier meta-analyses in non-valvular atrial fibrillation showed that OAC reduce ischemic stroke by ∼50%–70% compared with no therapy or aspirin, underscoring the superior efficacy of anticoagulation over antiplatelet therapy (10). Further, systematic reviews of direct oral anticoagulants (DOACs) vs. VKAs have demonstrated at least comparable efficacy in stroke prevention and often improved safety, particularly with respect to intracranial hemorrhage and all-cause mortality (11). DOACs have been shown to reduce stroke or systemic embolism more effectively than warfarin, while major bleeding outcomes were lower or comparable in many pooled analyses (12). The evidence comparing anticoagulants vs. antiplatelets also supports our findings: anticoagulant therapy in acute ischemic stroke patients with atrial fibrillation was associated with significantly lower mortality and intracranial hemorrhage compared with antiplatelet therapy (13).

Moreover, antiplatelet therapy consistently ranked lower for ischemic prevention, consistent with previous evidence (10). OAC strategies demonstrated higher efficacy for stroke reduction, as expected given the dominant cardioembolic mechanism in post-repair/TEER patients and their overall protective effect against stroke (10, 12). These findings mirror trends in large atrial fibrillation meta-analyses and TEER-focused observational data (11). The resulting hierarchy, anticoagulant strategies (particularly DOACs) outperforming antiplatelet regimens for bleeding and mortality, appears clinically plausible and aligns with guideline-driven priorities for anticoagulation in high-embolic-risk patients. At the same time, the need to balance efficacy and safety remains critical: while anticoagulants reduce ischemic outcomes and death, bleeding risks must be managed carefully, particularly in vulnerable or post-repair populations. Future research should further refine patient selection and procedural contexts to optimize this balance.

Several design and clinical factors clarify why effects differ across studies and, consequently, why our pooled and ranked estimates look the way they do. First, cohorts varied when follow-up began, and which windows were emphasized. Some excluded peri-procedural events (where bleeding predominates), whereas others included the early phase or focused on mid-term horizons (when thromboembolism becomes more salient). Such windowing naturally shifts the apparent net benefit of one regimen over another (4). Second, peri-procedural management differed (e.g., heparin bridging vs. no bridging). Randomized evidence shows that forgoing warfarin bridging lowers bleeding without increasing thromboembolism; centers that adopt bridging more liberally would therefore make VKA-based strategies look worse on bleeding, moving DOACs up in the rankings (13).

Pharmacology and quality of anticoagulation also mattered. Several European datasets use phenprocoumon rather than warfarin; phenprocoumon's much longer half-life can yield steadier inhibition and different bleeding/INR dynamics than warfarin, which may compress differences vs. DOACs in those settings and expand them elsewhere (14). Among VKA users, time in therapeutic range (TTR) is rarely reported yet strongly linked to outcomes; centers with low TTR will make DOACs look superior for both bleeding and stroke, whereas high-TTR centers attenuate advantage-precisely the effect-modification seen in RE-LY analyses (15).

Concomitant antiplatelet therapy adds another layer. A sizable fraction of post-TEER patients receives OAC plus one (or even two) antiplatelet agents because of prior PCI, coronary disease, or local protocols. Modern randomized AF evidence shows that adding aspirin to OAC increases bleeding with little additional ischemic benefit when a P2Y12 inhibitor is used, so sites with heavier antiplatelet co-therapy will inflate bleeding rates in the OAC arms and degrade their ranking relative to DOAC-only use (16). Relatedly, our network likely absorbed off-label DOAC dose reductions in frail/elderly patients; such underdosing is associated with more thromboembolism and inconsistent bleeding gains, which would blunt DOACs' apparent stroke advantage in some nodes of the network (17). Renal dysfunction, a common comorbidity after mitral interventions, also modifies DOAC–VKA trade-offs and contributes to between-study dispersion (18).

Outcome ascertainment and definitions further explain discrepancies. Claims-based studies may misclassify bleeding and stroke compared with adjudicated registries (19). Included cohorts used different bleeding taxonomies: ISTH, BARC, and TIMI (20). These definitional shifts change event counts and perceived severity and thus alter comparative estimates and rankings (21). Finally, because our evidence base is predominantly nonrandomized, confounding by indication is inevitable: clinicians preferentially assign antiplatelet therapy to patients judged “too fragile to anticoagulate” (22) and VKAs to those with specific indications (e.g., mechanical valves, high thrombotic risk), whereas DOACs are often selected for perceived safety (23). Such selection patterns bias crude contrasts even after adjustment and are a central reason why net-work estimates can shift when node composition changes across studies.

Two additional context points help interpret why our network landed where it did. Guideline and consensus documents around structural heart disease and surgical repair commonly endorse an initial VKA period after surgical repair, whereas post-TEER antithrombotic regimens are less standardized. Centers that adhere to early VKA after open repair but individualize therapy after TEER will seed network with systematically different patients in VKA vs. DOAC nodes, amplifying heterogeneity and sometimes reversing the direction of apparent benefit across subgroups (24). And at the method level, irregular networks with sparse or unbalanced comparisons can bias rank probabilities; our interpretation therefore emphasizes effect sizes and uncertainties alongside rankings to avoid over-reading hierarchy signals from asymmetric evidence (25).

Taken together, variations in biological mechanisms, timing of outcome assessment, peri-procedural management, VKA type and time in therapeutic range (TTR), concomitant antiplatelet use, dose selection, comorbidities, outcome definitions, and nonrandomized treatment allocation collectively explain both the overall consistency and residual variability observed across studies and network nodes. These factors help to clarify why our main findings consistently favored OAC over antiplatelet therapy, and why DOACs generally appeared safer than VKAs for bleeding while providing comparable stroke protection. Moreover, they illustrate how pragmatic treatment choices and real-world practice patterns likely influenced the specific ranking distributions observed in our network meta-analysis. Importantly, treatment ranking probabilities derived from sparse networks can be unstable and sensitive to small changes in the evidence base; therefore, the ranking results presented in this study should be viewed as exploratory indicators rather than definitive guidance for clinical decision-making. Differences between phenprocoumon and warfarin across regions, including longer half-life and potential differences in time-in-therapeutic-range, may partly explain the observed estimates and highlight the need for cautious interpretation when comparing VKAs across heterogeneous healthcare settings. Because antiplatelet therapy was analyzed as a single aggregated category encompassing both SAPT and DAPT regimens with variable durations, the observed estimates may reflect treatment heterogeneity rather than the effect of a single antiplatelet strategy. Therefore, these findings should not be interpreted as evidence against all antiplatelet approaches in every clinical context.

### 4.1 Limitations

Several limitations temper the certainty of these conclusions. First, all included studies were observational, with inherent susceptibility to selection bias and residual confounding despite statistical adjustments. Second, the heterogeneity in comparator drugs, especially the use of phenprocoumon in European cohorts vs. warfarin in U.S. cohorts, introduces regional variability that complicates pooled inference. Third, outcome definitions were inconsistent across studies (ISTH, BARC, TIMI), which may have altered event classification and comparability. Additionally, concomitant antiplatelet use, variable dosing (including off-label DOAC reductions), and differing follow-up windows further limit cross-study uniformity. Finally, the evidence network remains sparse and irregular, with most contrasts informed by only one or two studies, reducing precision and amplifying the potential for biased ranking estimates. These limitations emphasize the need for prospective, randomized studies to definitively guide antithrombotic management after TMVR/TEER. Fourth, APT included heterogeneous regimens (SAPT vs. DAPT, differing durations), so the observed inferiority of APT cannot be applied to all antiplatelet strategies. Fifth, major bleeding was defined using different classification systems across studies (including ISTH, BARC, and TIMI definitions), which may affect the comparability of pooled estimates and introduce heterogeneity into the analysis. Although this heterogeneity was unavoidable given the limited available evidence, it should be considered when interpreting the magnitude of bleeding risk estimates across treatment groups.

## 5 Conclusions

This meta-analysis provides the first comprehensive synthesis of antithrombotic strategies in patients undergoing TMVR/TEER with atrial fibrillation. OAC strategies appeared to be associated with lower bleeding risk and potentially favorable outcomes compared with antiplatelet therapy, although the available data were insufficient to draw firm conclusions regarding stroke prevention. Mortality signals were less consistent, but DOAC tended toward better mortality outcomes compared to Warfarin in some contexts. Taken together, these findings highlight OAC, particularly DOACs, as the most balanced strategy for reducing thromboembolic risk and potential mortality benefits without a prohibitive bleeding penalty, underscoring their potential role as the preferred regimen in this high-risk population.

## Data Availability

The original contributions presented in the study are included in the article/Supplementary Material, further inquiries can be directed to the corresponding author.
