# Supplementary material for: Efficacy and safety of different antithrombotic treatment regimens in patients undergoing transcatheter mitral valve repair and atrial fibrillation; A meta-analysis and systematic review
Source: Front Cardiovasc Med. 2026 Jul 21;13:1743097. doi: 10.3389/fcvm.2026.1743097 (PMC13433443; doi:10.3389/fcvm.2026.1743097)
Supplement: Supplementary file 1 [file Table1.docx]

**Table S1. STROBE checklist**

| **Item (STROBE No.)** | **Recommendation** | **Schipper et al. (2025)** | **Wächter et al. (2022)** | **Hohmann et al. (2022)** | **Mentias et al. (2022)** |
| --- | --- | --- | --- | --- | --- |
| **Title/Abstract** | Indicate design in title/abstract | Title specifies “observational study” | Abstract describes multicenter observational cohort | Abstract specifies “retrospective observational study” | Abstract specifies “observational Medicare cohort” |
| **Background** | Scientific background, objectives | Clear rationale for DOAC vs VKA after M-TEER | Rationale for ATT variation post-TEER | Justification for real-world anticoagulation practice | Objective to compare DOAC vs warfarin in valve AF |
| **Study Design** | Present key design elements early | Retrospective, single-center registry | Multicenter retrospective cohort | Retrospective claims database | National Medicare observational cohort |
| **Setting** | Location, setting, dates | Cologne, Germany; 2012–2019 | 4 German tertiary centers; 2011–2021 | German claims database; 2014–2018 | U.S. Medicare data; 2015–2019 |
| **Participants** | Eligibility, selection, follow-up | AF patients post-M-TEER, consistent OAC | All TEER patients, stratified by ATT | Patients receiving MitraClip in claims database | AF patients post-valve repair/replacement |
| **Variables** | Clearly define outcomes, exposures | DOAC vs VKA; outcomes: ischemic, bleeding, death | OAC mono, OAC+APT, DAPT; mortality, MACCE | OAC, APT, combination; mortality, MACE, bleeding | DOAC vs warfarin; mortality, stroke, bleeding |
| **Bias** | Address bias | Excluded APT to reduce confounding; adjusted analysis | Multivariate Cox regression; lost-to-follow-up <6% | Claims-based definitions, risk of misclassification | Propensity score/IPW adjustment to limit confounding |
| **Study Size** | Explain size | N=206 (OAC=61, VKA= 145) | N=609 (OAC=146, APT=149) | N=1342 (APT=157, OAC=279) | N=1178 (MV repair cohort) (OAC=491, WAR-687) |
| **Quantitative Variables** | How handled | Continuous as mean±SD; categorical as % | Stratified ATT groups, survival models | Claims data grouped by therapy | Weighted survival analysis with IPW |
| **Statistical Methods** | Detail methods | HR, Cox regression, multivariable adjustments | Cox regression, regression models | Cox regression, HR, adjusted models | IPW Cox regression, sensitivity PS-matching |
| **Results** | Participants, descriptive data, outcomes | Kaplan–Meier, HR, mortality differences | ATT distribution Sankey, mortality HR | Regimens distribution, adjusted HR | Mortality, stroke, bleeding HR |
| **Discussion** | Key results, limitations, interpretation | Notes observational design, lack RCTs | Acknowledge heterogeneity, need RCTs | Highlights heterogeneity, no guideline clarity | Notes limitations of Medicare data, observational |
| **Other Info** | Funding, ethics, conflicts | Ethics approved, funding disclosed | Ethics approval, multicenter | Claims database, no patient contact, funding noted | CMS data, IRB approval, disclosures |
