# Supplementary material for: Efficacy and safety of different antithrombotic treatment regimens in patients undergoing transcatheter mitral valve repair and atrial fibrillation; A meta-analysis and systematic review
Source: Front Cardiovasc Med. 2026 Jul 21;13:1743097. doi: 10.3389/fcvm.2026.1743097 (PMC13433443; doi:10.3389/fcvm.2026.1743097)
Supplement: Supplementary file 2 [file Table2.docx]

**Table S2. Baseline characteristics**

| **Study Name** | **Age (APT / OAC / VKA / Warfarin)** | **Female (APT / OAC / VKA / Warfarin)** | **Hypertension (APT / OAC / VKA / Warfarin)** | **Stroke (APT / OAC / VKA / Warfarin)** | **Diabetes (APT / OAC / VKA / Warfarin)** | **Peripheral vascular disease (APT / OAC / VKA / Warfarin)** | **Chadsvasc score (APT / OAC / VKA / Warfarin)** |
| --- | --- | --- | --- | --- | --- | --- | --- |
| **Mentias 2022** | 77.5±9.2 (APT) 75.0±8.7 (VKA) | 49.5 (APT) 47 (VKA) | 87.6 (APT) 85 (VKA) | 9.4 (APT) 8.2 (VKA) | 25.3 (APT) 27.8 (VKA) | 19.1 (APT) 18.1 (VKA) | 5 (4–6) (APT) 5 (4–6) (VKA) |
| **Hohmann 2022** | 74.6 (10.4)(APT) 77.0 (8.2) (OAC) | 34.4 (APT) 41.8 (OAC) | 85.4 (APT) 93.1 (OAC) | 12 (APT) 24 (OAC) | 74 (APT) 126 (OAC) |  | 4.3 (APT) 4.7 (OAC) |
| **Waechter 2022** | 78.5 ± 7 (APT) 78.3 ± 7.5 (OAC) | 46.8 (APT) 41.1 (OAC) | 80.3 (APT) 87.1 (OAC) | 11 (APT) 14 (OAC) | 46 (APT) 46 (OAC) |  |  |
| **Schipper 2025** | 78.4±7.4 (OAC) 78.6±7.9 (VKA) | 49 (OAC) 45 (VKA) | 74 (OAC) 69 (VKA) | 13 (OAC) 22 (VKA) | 13 (OAC) 37 (VKA) | 5 (OAC) 11 (VKA) |  |
